# The evaluation of global cognitive and emotional status of older patients with chronic tinnitus

**DOI:** 10.1002/brb3.2074

**Published:** 2021-07-21

**Authors:** Anna Rita Fetoni, Tiziana Di Cesare, Stefano Settimi, Bruno Sergi, Giorgia Rossi, Rita Malesci, Camillo Marra, Gaetano Paludetti, Eugenio De Corso

**Affiliations:** ^1^ Institute of Otolaryngology Fondazione Policlinico Agostino Gemelli IRCCS Rome Italy; ^2^ Università Cattolica del Sacro Cuore Rome Italy; ^3^ Audiology and Vestibology Unit Neuroscience Department Federico II University Naples Naples Italy; ^4^ Memory Clinic Fondazione Policlinico Agostino Gemelli IRCCS Rome Italy

**Keywords:** cognitive impairment, older patients, psychiatric disorders, tinnitus

## Abstract

**Objectives:**

Tinnitus is a common symptom largely impactful on quality of life, especially in the elderly. Our aim was to evaluate the efficacy of self‐administered screening tests to correlate the severity of subjective perception of tinnitus with emotional disorders and the overall cognitive status.

**Methods:**

Patients aged ≥ 55 years with chronic tinnitus were recruited and submitted to a complete audiological evaluation; Tinnitus Handicap inventory (THI); Hospital Anxiety and Depression Scale (HADS‐A and HADS‐D) and Mini‐Mental State Examination (MMSE). Demographic and audiological features of patients with and without cognitive impairment (MMSE score cut‐off of 24/30) were analyzed in order to reveal the relationship among tinnitus, emotional disorders, and cognitive dysfunction.

**Results:**

102 patients were recruited (mean age: 70.4 ± 9.6). THI score was directly related to HADS‐A score (*r* = .63) HADS‐D score (*r* = .66), whereas there was no relationship between tinnitus severity and MMSE (*r* = .13). CI and n‐CI groups did not differ in the characteristics of tinnitus (*p* > .05), however, hearing threshold (*p* = .049) and anxious depressive traits measured with HADS‐A (*p* = .044) and HADS‐D (*p* = .016) were significantly higher in the group with cognitive impairment. Furthermore, age ≥ 75 years (*p* = .002, OR = 13.8), female sex (*p* = .032; OR = 6.5), severe hearing loss (*p* = .036; OR = 2.3), and anxiety (*p* = .029; OR = 9.2) resulted risk factors for CI. Therefore, in CI group MMSE score was inversely related to age (*r* = −.84).

**Conclusions:**

Cognitive impairment and psychiatric discomfort should be considered in tinnitus patients, related to increasing age, female sex, and severe hearing loss. Thus, self‐administered questionnaires can be useful in addressing clinical approach.

## Graphical abstract

Anxious/depressive traits are higher prevalent in geriatric tinnitus patients. Risk of cognitive dysfunction increases in relation to increasing age, female sex and severe hearing loss. Aging is an independent factor for the development of cognitive dysfunction in tinnitus‐suffers as well as severe hearing loss; Self‐administered screening tests for global cognitive and emotional status are recommended in managing of older patients with tinnitus.

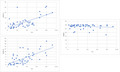

## 1 INTRODUCTION

Tinnitus, consisting in the perception of sounds in the absence of an external stimulus, is a very common and disabling condition with pervasive effects on health and wellbeing, substantive economic burden, and no known cure. About 20%–30% of people in industrialized societies are mildly affected by chronic tinnitus, and some of them report tinnitus severely influencing their daily life (Bhatt et al., 2016; Gallus et al., 1998). Prevalence increases with age, ranging from 24% to 45% in the elderly (Sindhusake et al., 2003), frequently accompanied by hearing impairment.

Tinnitus could have an heterogeneous clinical presentation; it can occur as an isolated idiopathic symptom or in association with any type of deafness, even if hearing threshold could be normal as in case of toxicity from aspirin or quinine (Ralli et al., 2010).

It could become a highly disabling symptom when it acquires an emotive significance through cognitive processes, making patients at risk of developing several disorders, such as insomnia and concentration problems, anxiety, and depression (McKenna et al., 2014). On the other side, many patients are able to tolerate this condition, showing a “hardiness” or “resilience” making them able to cope with this kind of stress (Wallhäusser‐Franke et al., 2014). Understanding why some people suffer and others do not is challenging in tinnitus research and clinical practice. The impact of tinnitus and the resulting changes in behavior have a central role in creating and maintaining distress. It is largely demonstrated that psychiatric discomfort is present in a large number of tinnitus‐suffers, with a higher prevalence of anxiety rather than depression (Cho et al., 2013; Fetoni et al., 2016). Psychiatric comorbidities play a major role in determining inter‐individual differences in subjective perception and tolerance of tinnitus, which cannot only be explained by the psychoacoustic measures of tinnitus (i.e., frequency and loudness) or by the associated hearing impairment (Pattyn et al., 2016).

Studies on animals and humans support the notion that tinnitus is related to a failure of the central auditory pathway to adapt to a critical loss of afferent peripheral fibers secondary to peripheral damage (Fetoni et al., 2015; Knipper et al., 2013), leading to plastic neuronal changes in the tonotopic map of the auditory cortex, also known as “maladaptive plasticity,” which concurs in maintaining tinnitus in a sort of “vicious circle” (Stolzberg et al., 2011). Cortical reorganization is accompanied by deprivation of excitatory and inhibitory inputs from the nuclei of the afferent auditory pathway, causing down‐regulation of inhibition and increase of excitability in several subcortical nuclei and in the auditory cortex (Thai‐Van et al., 2007).

It has been experimentally emphasized the role of hyperactivity in the peripheral auditory system, especially the dorsal cochlear nucleus and inferior colliculus, in generating the “bottom‐up” tinnitus percept. In this context, conscious perception of tinnitus may result by the failure of top‐down mechanisms, such as attention switching, to inhibit this bottom‐up hyperactivity. Therefore, neurocognitive dysfunction in both auditory and nonauditory pathways might explain the increased psychological comorbidities related to the tinnitus perception (Trevis et al., 2017).

All these changes in the central auditory pathway, together with the neuroplastic reorganization within the auditory cortex, thalamus, and the structures of the limbic and paralimbic circuits (Rauschecker et al., 2010) induced some Authors to speculate about a possible relationship between tinnitus and cognitive impairment (CI), with a positive correlation to tinnitus severity (Araneda et al., 2015; Wang et al., 2018).

Consequently, patients experiencing severe tinnitus may suffer higher cognitive deficits with obvious decrease in quality of life and work productivity (Wang et al., 2018), which have been excluded in other reports (Waechter & Brännström, 2015). However, it is clinically well known that in some patients tinnitus lead to frustration and difficulty concentrating (Burton et al., 2012) affecting cognitive functions. It has been recently showed that psychological markers of attention‐switching, namely cognitive and emotional control, are impaired in chronic tinnitus (Trevis et al., 2016; Uchida et al., 2019); however, the relationship among these features and cognitive decline in older patients is controversial. Despite tinnitus could affect people with normal audiometric threshold, the connection between tinnitus sensation pitch and hearing loss is well established mainly in older patients with high‐frequency hearing loss (Schecklmann et al., 2014; Schilder et al., 2014). Conversely, presbycusis and age‐related tinnitus can affect mental health and contribute in developing anxiety, stress, and depression. Consequently, it is difficult to understand whether nonauditory effects are caused by tinnitus rather than hearing loss. Thus, further studies are needed to clarify the distinct contribution of tinnitus, hearing loss, and their comorbidities in developing cognitive decline in physiological aging and in predisposing the landmarks of dementia. Growing evidences suggest dependent effects of age‐related hearing loss (ARHL) on risk for late‐life cognitive decline and depression (Rutherford et al., 2018). Increased listening effort due to hearing deprivation may lead older adults to avoid social interaction, exacerbating loneliness, depression, and reducing well‐being. Depression and social isolation may mediate in turn the relationship between hearing loss and cognitive impairment (Uchida et al., 2019). Thus, tinnitus that co‐occurs with ARHL can independently lead to increased risk of depression and cognitive dysfunction (Fetoni et al., 2016; Shargorodsky et al., 2010). For this reason, the management of tinnitus and ARHL must involve integrated care that considers the individual's entire audiological and neurocognitive profile.

Screening for anxious/depressive traits and CI may be indicated among older patients with tinnitus to identify individuals at high‐risk for adverse outcomes. Given the above, this research addresses the application of questionnaires useful to assess the global cognitive and emotional status of older patients suffering from chronic tinnitus administered during audiological counseling to face further audiological and neurocognitive assessment toward a personalized therapeutic approach.

## 2 MATERIALS AND METHODS

This was an observational study of patients admitted for chronic tinnitus at the department of Otolaryngology of our Institution, from September 2019 to February 2020. Before enrollment, all patients received complete and comprehensible information about the tests administered and gave their written consent to their execution, according to the Ethical Committee recommendation, in agreement with the ethical standards of the Declaration of Helsinki.

Inclusion criteria were as follows: Age ≥ 55 years that is considered at a risk for the onset of cognitive impairment (Petersen, 2004); history of chronic tinnitus (longer than 3 months); and the ability to read, understand, and answer to the assigned questionnaires and to give written informed consent. We will consider eligible for the study both patients with and without diagnosis of neurosensory hearing loss at standard tonal audiometry.

We excluded patients affected by acute and chronic inflammatory disease of the middle ear, otosclerosis, patients with inner ear diseases (particularly Meniere's disease), objective tinnitus, and tinnitus caused by abnormal anatomical structure of the external and middle ear. Patients with neurodegenerative disease (especially Alzheimer's and Parkinson's disease), known history of neuropsychiatric or brain disorder, and patients using psychotropic or central nervous system‐active medications were also excluded considering that neurocognitive diseases and treatments should be a confusion in relation to the occurrence of emotional/cognitive dysfunction related to tinnitus. Alcohol and drug abuse was also exclusion criteria.

We performed a complete audiological entry‐level evaluation to all patients including the following: otoscopy, in order to determine the integrity of the conductive mechanism; tympanometry and acoustic reflex measurement (Grason Stadler Tympstar); and standard pure‐tone audiometry, testing conventional frequency ranging from 0.25 to 8 kHz (Amplaid 319 audiometer—Amplaid Inc.) in a double‐walled, soundproof room, including psychoacoustic tests of tinnitus, such as tinnitus pitch and loudness matching. Pure‐tone average (PTA) value was calculated as the mean of 0.25, 0.5, 1, 2, and 4‐kHz thresholds.

For what concerns the evaluation of tinnitus severity, we administered the validated Italian version of the *Tinnitus Handicap Inventory* (THI) (Passi et al., 2008). THI is a questionnaire composed by 25 items divided in three subgroups: The functional scale (11 items) concerns the discomfort experimented by the patient in the cognitive, social, working and physic areas; the emotional scale (nine items) includes a wide range of emotions generated by the tinnitus; and the third subgroup (five items) reflects the catastrophic situation of the patient who cannot cope with this disorder, feeling hopeless, and affirming to be affected by a terrible disease. For each item, the patient can assign a value of 0, 2, or 4 points, depending on whether or not he fits himself in the described setting. The final score is used to assign the subject to one of the five categories characterized by rising level of distress. THI proved to be an affordable and reliable tool to evaluate the gravity of tinnitus subjective perception in heterogeneous populations.

The prevalence of emotional disorders was evaluated by the validated Italian version of *Hospital Anxiety and Depression Scale* (HADS) (Costantini et al., 1999), a tool of 14 questions addressed to nonpsychiatric outpatient clinics, in which an emotional assessment is required to improve the diagnosis and try a psychological therapeutic approach. HADS is divided in two subgroups of seven questions: the HADS‐A, specific for anxious disorder, and the HADS‐D, specific for depressive disorder. Each item of the questionnaire is scored from 0 to 3 (total final score between 0 and 21 for either anxiety or depression). In both subscales, the total score of eight out of 21 indicates the cutoff point for anxiety or depression.

Finally, a comprehensive cognitive evaluation was carried out by administering the Italian version of *Mini‐Mental State Examination* (MMSE) (Franco‐Marina et al., 2010), a 30‐point questionnaire for detecting the prevalence of cognitive impairment. Administration of the test takes between 5 and 10 min, and it is composed by items referring to eight different cognitive areas: orientation to place, orientation to time, registration, attention and calculation, recall, language, repetition, and complex commands. Any score of 24 or more (out of 30) indicates a normal cognition. Below this, scores can indicate severe (≤9 points), moderate (10–18 points), or mild (19–23 points) cognitive impairment. Consequently, CI group was defined as MMSE score cutoff of 24 (Guglielmi et al., 2020).

Statistical analysis was performed using SPSS 25 for Windows (Chicago, Illinois). Continues values such as symptoms scores were expressed as mean ± standard deviation (*SD*). To analyze the association between tinnitus severity (based on THI score) and cognitive deficits (based on MMSE score) as well as between tinnitus and psychiatric disorders (based on HADS scores), multiple Pearson's correlation analysis were performed and Bonferroni's correction for multiple comparisons was applied, considering correlation significant for *p* value < .01.

To determine significant differences between patients with and without cognitive impairment (CI) in the screened demographic and audiological continuous variables, independent Student's *t* tests were performed. We used regression analysis to assess potential risk factors for cognitive impairment in tinnitus patients. The results were considered significant for *p* values < .05.

## 3 RESULTS

We recruited 102 patients suffering from chronic tinnitus for an average of 47.4 months (range = 4–240 months). Mean age was 70.4 ± 9.6 years (range 55–94 years); male‐to‐female ratio was 0.7. Most of the sample (71/102, 69.6%) reported bilateral tinnitus, while 31.4% reported it unilaterally.

**FIGURE 1:**
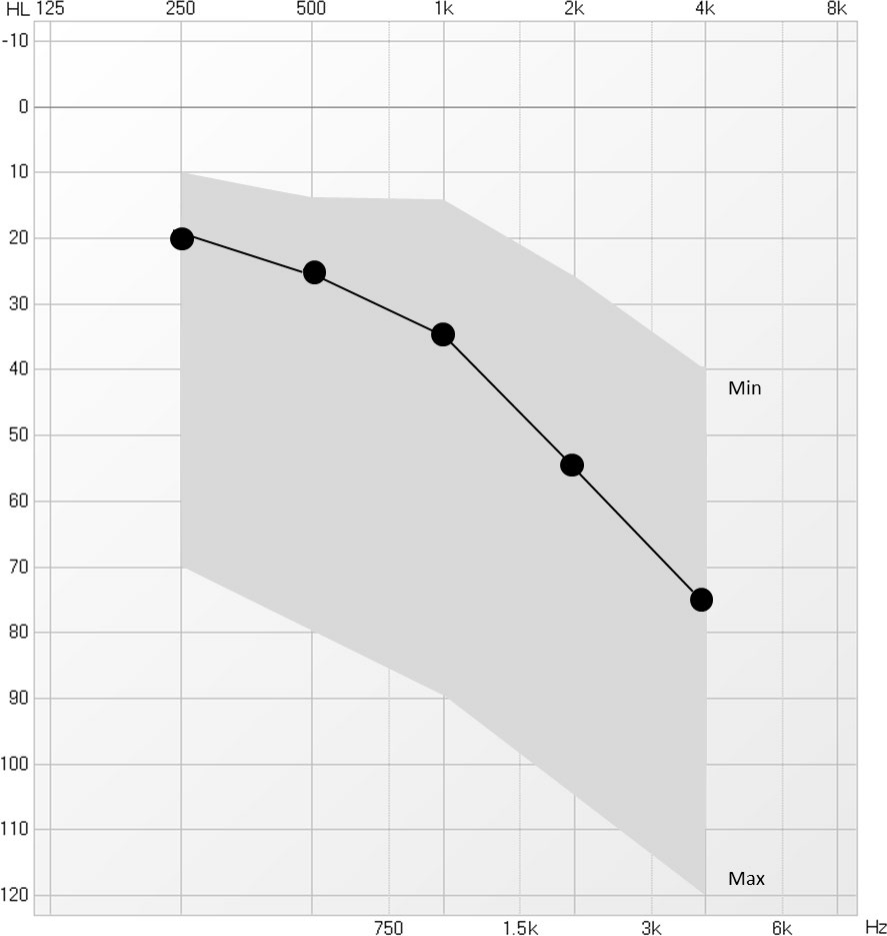
The average audiogram of both ears in our sample (mean PTA = 39.7 ± 22.65 dB HL); min: lowest observation; max: highest observation

**FIGURE 2:**
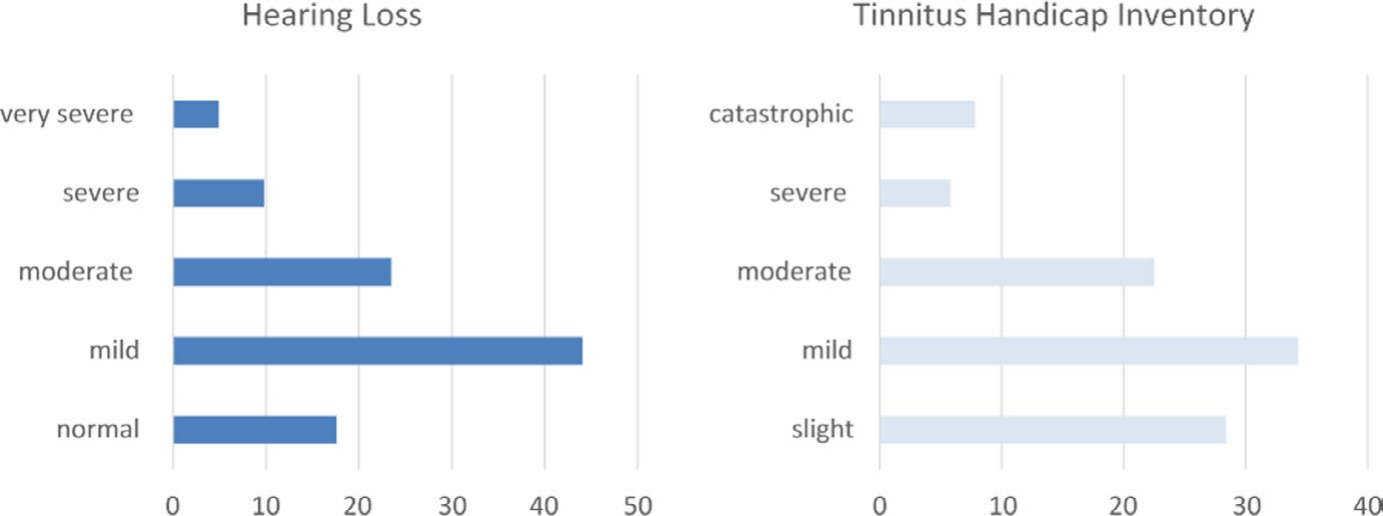
Histograms display percentage distribution of patients in the different levels of severity of hearing loss and tinnitus perception

Tonal audiometry results showed that the mean value of PTA of both ears was 39.7 ± 22.65 dB HL (range = 13–113 dB HL). The typical audiogram shape was a bilateral, symmetric, high‐frequency (i.e. 4–8 kHz) hearing loss (Figure 1). Namely, 45/102 (44.1%) patients presented mild hearing loss (PTA between 21 and 40 dB HL); 24/102 (23.5%) moderate hearing loss (PTA between 21 and 60 dB HL); 10/102 (9.8%) severe hearing loss (PTA between 61 and 90 dB HL); and 5/102 (4.9%) very severe hearing loss (PTA higher than 90dB HL). 18/102 patients (17.6%) had a normal‐hearing threshold (equal or below 20 dB HL) (Figure 2).

Average score obtained by THI was 32.8 ± 22.5, which stands for “mild” degree of discomfort. Tinnitus‐induced discomfort, in particular, was reputed “slight” by 30/102 (28.4%), “mild” by 35/102 (34.3%), “moderate” by 23/102 (22.5%), “severe” by 6/102 (5.8%), and “catastrophic” by 8/102 (7.8%) (Figure 2).

The HADS questionnaire revealed the overall emotional disorders in 47/102 (46%) patients affected by tinnitus. Namely, 21/102 (20.6%) patients suffered from anxiety symptoms, 26/102 (25.5%) patients were affected by depressive symptoms; and anxiety and depression were present in combination in 10/102 (9.8%) patients.

**FIGURE 3:**
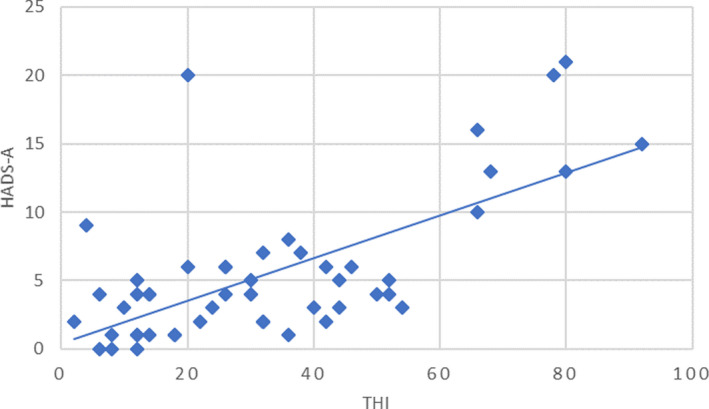
Relationship between THI score and HADS‐A score; Pearson's *r* = .63

**FIGURE 4:**
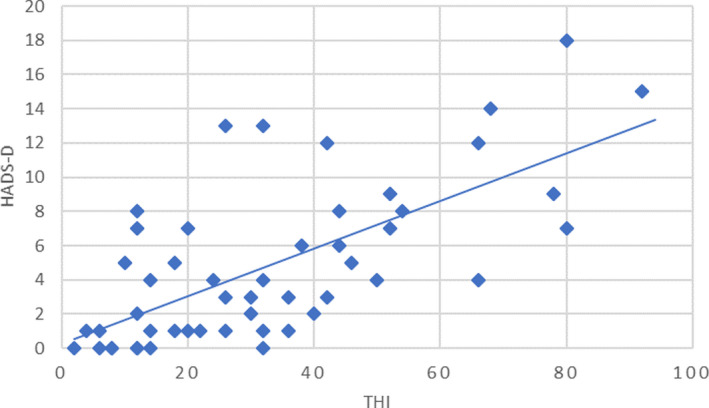
Relationship between THI score and HADS‐D score; Pearson's *r* = .66

A statistically significant correlation between the THI and the HADS‐A score (*r* = .63, *p* < .0001) (Figure 3) and between the THI and the HADS‐D score (*r* = .66, *p* < .0001) (Figure 4) was demonstrated by the Pearson's correlation test in the total sample. Thus, the severity of tinnitus was related to the presence of psychiatric discomfort.

The mean score of MMSE used as a screening test for CI was 27.7 ± 3. Most of the sample, 85/102 (83.3%) patients, obtained a score ≥ 24 (normal condition); 17/102 (16.6%) patients showed some degree of CI (score < 24). Particularly, 14/102 (13.7%) patients presented a mild CI (score 19–23), and 3/102 (2.9%) had a moderate CI (score 10–18). None of the present sample had a severe degree of cognitive impairment.

**FIGURE 5:**
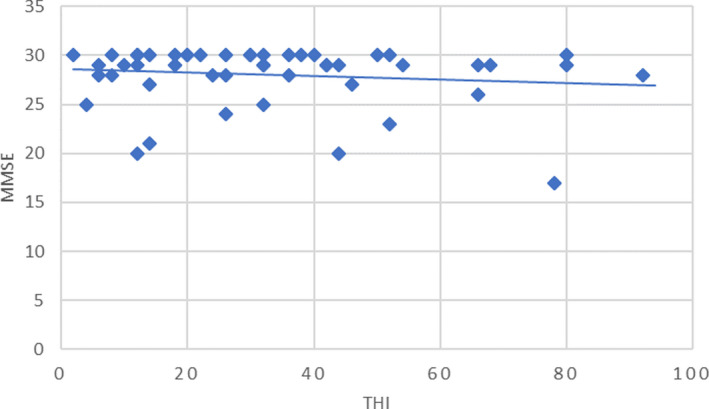
Relationship between THI score and MMSE score; Pearson's *r* = .13

We did not find a significant correlation between the neuropsychological discomfort measured with THI and the score obtained at the MMSE (*r* = .13; *p* > .05) indicating that there was not an overall relationship between the severity of tinnitus and cognitive impairment (Figure 5). Demographic and clinical characteristics of CI group were analyzed and compared with the other tinnitus‐suffering patients (n‐CI group) in order to reveal the relationships between cognition and possible factors influencing cognitive abilities (Table 1, Table 2). Age of patients and tinnitus features (duration, pitch, loudness, and THI score) with respect to the decreased values of MMSE were not statistically different (*p* > .05) (Table 1).

**TABLE 1:** Demographics and clinical features of the patients in the cognitive impairment group (CI) and noncognitive impairment group (n‐CI); intergroup comparison performed with *t* test for unpaired data

	CI group (MMS < 24) *n* = 17	*n*‐CI group (MMS ≥ 24) *n* = 85	*p* value
Age (years; mean ± *SD*)	76.2 ± 10	69.2 ± 9.6	>.05
Audiological results			
Pure Tone Average (dB; mean ± *SD*)	49.6 ± 23.4	37.5 ± 23.4	.0490
Tinnitus features			
Tinnitus duration (months; mean ± *SD*)	55.8 ± 53.5	64.3 ± 50.7	>.05
Tinnitus pitch (kHz; mean ± *SD*)	4.1 ± 0.5	4.5 ± 0.6	>.05
Tinnitus loudness (dB; mean ± *SD*)	55.0 ± 20	49.2 ± 22	>.05
THI score (mean ± *SD*)	32.7 ± 23.4	32.8 ± 22.3	>.05
Psychiatric disorders			
HADS‐A score (mean ± *SD*)	8.1 ± 5.4	5.2 ± 5.3	.0440
HADS‐D score (mean ± *SD*)	7.6 ± 4.9	4.6 ± 4.7	.0160

**TABLE 2:** Regression analysis and risk factors for cognitive impairment

	SE	Wald	p value	OR	Lower limit 95% CI	Upper limit 95% CI
Female sex	0.875	4.617	.032	6.559	1.180	36.466
Age ≥ 75 years	0.859	9.359	.002	13.865	2.572	74.733
THI ≥ 58	1.456	0.710	.301	0.042	0.002	2.736
Tinnitus duration ≥60 months	0.973	0.129	.719	0.705	0.105	4.747
HADS‐A ≥ 8	1.099	4.104	.029	9.274	1.375	80.011
HADS‐D ≥ 8	0.899	0.031	.860	1.172	0.201	6.832
Severe/very severe hearing loss	0.935	3.838	.036	2.352	1.377	14.687

Interestingly, regression analysis showed higher risk of cognitive impairments in females (*p* = .032; OR = 6.5) and older patients ≥ 75 years old (*p* = .002; OR = 13.8) (Table 2). In CI group, MMSE was inversely related to age (*r* = −.84); thus, a relationship was found among tinnitus and CI in older patients indicating that aging is an independent factor for the development of CI in tinnitus‐suffers.

With regard to hearing abilities, patients with CI had lower hearing threshold levels (PTA of both ears, *p* = .049) (Table 1); particularly 41.2% in CI group versus 9.4% in n‐CI group had a severe or very severe hearing loss (PTA > 60dHL). Degraded listening skills represent a risk factor for CI in patients with tinnitus (*p* = .036; OR = 2.3) (Table 2).

Furthermore, CI group totalized higher scores with HADS‐A (*p* = .044) and HADS‐D (*p* = .016) questionnaires (Table 1). Anxiety was found in 41.2% versus 16.5% in CI group and n‐CI group, respectively, showing that patients with tinnitus and anxious disorder had greater risk than not anxious ones of developing some degree of difficulty in cognitive skills (*p* = .029; OR = 9.2) (Table 2). Taken together, these data demonstrated that in tinnitus‐suffers, cognitive impairment, as detected by using MMSE, was found predominantly related to increasing age, female sex, and severe hearing loss.

## 4 DISCUSSION

Despite the widespread prevalence of tinnitus as one of the most common disorders especially in increasing age, there is little consensus on its underlying mechanisms and management approach. Herein, we assessed the degree of tinnitus annoyance in older patients suffering from chronic tinnitus in order to correlate the severity of tinnitus perception to the presence of anxious–depressive traits and CI, addressing patients to a multidisciplinary and personalized approach. Major findings are that anxious/depressive traits are higher prevalent in geriatric tinnitus patients with respect to cognitive impairment. However, the risk of cognitive dysfunction, as detected by MMSE, increased in older indicating that aging is an independent factor for the development of CI in tinnitus‐suffers as well as severe hearing loss.

Namely, our results demonstrate the overall prevalence of about 46% psychiatric comorbidity with a slight preponderance of depression with respect to anxiety (26 vs. 20% respectively). The higher prevalence of emotional disorders in patients suffering of more debilitating tinnitus suggests that a comorbid psychological disorder influences the perceived severity of tinnitus and quality of life. However, the causal link between tinnitus perception and psychological discomfort is still object of studies: On one hand, the emotional status derived from tinnitus may trigger the psychiatric disorder in patients at risk, and on the other tinnitus could unmask a pre‐existing compensated disorder. Current evidence suggests a complex association between tinnitus and depression (Martz et al., 2019) because a causal relationship should not be assumed considering the possibility of a reverse association in which depression trigger an exacerbation of tinnitus. Thus, tinnitus like chronic pain may prompt a depressive episode that may impede habituation to the aversive sensation increasing the severity of its perception (Salazar et al., 2019). However, neurobiological substrates of this bidirectional relationship are still a matter of debate.

Hoping that new insight on the interaction of a larger network including several forebrain circuits, the auditory cortex and the limbic structures such as the amygdala, anterior cingulate cortex, hippocampus, orbitofrontal cortex, and anterior insular cortex will explain a causal relationship (Langguth et al., 2012), currently the significant coexistence of tinnitus and psychiatric disorders and their neurobiological overlap support the recommendation to screen all patients with tinnitus for psychological distress using screening procedures.

Several screening scales have been introduced as tools to discriminate anxiety and depressive traits. The Hospital Anxiety and Depression Scale (Zigmond & Snaith, 1983) is a widely used 14‐item self‐report nonverbal questionnaire consisting of two subscales, one measuring anxiety (HADS‐A), the other measuring depression (HADS‐D). Among the other different scales, widely used is the Hamilton Anxiety Rating Scale (HAM‐A) to discriminate anxiety, the Centre for Epidemiology Studies Depression Scale (CES‐D) for the depressive traits alone or the BADI (Beck Anxiety and Depression Inventory) that is composed by 42 items. Some studies evaluated the correlation between these scales (Matza et al., 2010; Rogers et al., 2006); however, HADS has been reported as an effective measure in discovering patient's anxious–depressive traits with a relatively short number of items (Cieśla et al., 2016; Iani et al., 2014), preventing the patient from losing concentration or answer superficially. For these reasons, we preferred the use of HADS, rather than other psychometric scales. Furthermore, since tinnitus‐suffers usually do not show full‐blown but very nuanced psychiatric symptoms, leading slowly to an impaired quality of life (Cieśla et al., 2016), the use of a concise scale may be useful as a preliminary screening tool, especially in the geriatric population, to trace the following diagnostic approaches in a multi‐disciplinary strategy. HADS could be also helpful in managing therapeutic strategies, in consideration of the lack of a well‐tolerated, widely diffused and reliable drug therapy for tinnitus.

In attempting the association of tinnitus with behavioral effects, a bidirectional interference between tinnitus and cognition has been suggested. While the cognitive and perceptual load probably has a significant influence on both the perception of tinnitus and its impact on emotional well‐being, on the other hand the overload of cognitive resources in chronic tinnitus leads to failures in daily activities and in the executive control tasks in accordance with the so‐called “load theory” (Khan & Husain, 2020). Present study aimed to investigate the association between tinnitus severity and cognitive function among aged patients with chronic tinnitus by using a simple and reliable nonverbal cognitive screening test such as the MMSE. Herein, overall MMSE scores did not correlate with the tinnitus severity in elderly patients aged > 55 years as measured with THI.

However, hearing loss and the overall emotional distress associated with tinnitus could contribute to poor concentration and cognitive difficulties. In fact, the comparison of results between patients with and without cognitive discomfort by means of MMSE showed that the two groups did not differ in the characteristics of tinnitus or its severity at THI (*p* > .05), but in hearing threshold (*p* = .049) and anxious depressive traits measured with HADS‐A (*p* = .044) and HADS‐D (*p* = .016), significantly higher in the group with cognitive impairment. Furthermore, age ≥ 75 years (*p* = .002, OR = 13.8), female sex (*p* = .032, OR = 6.5), and anxiety (*p* = .029, OR = 9.2) resulted risk factors of cognitive discomfort in tinnitus patients. Therefore, in CI group MMSE score was inversely related to age (*r* = .84). Interestingly, severe hearing loss increased the risk of development signs of cognitive disorders (*p* = .03; OR = 2.36).

There is a large consensus considering auditory deprivation as a trigger of a vicious circle in older people, involving social isolation, quality of life deterioration, depression, and CI (Fabijańska et al., 2012; Fortunato et al., 2016). It is widely accepted that ARHL is an independent and modifiable risk factor for dementia (Anzivino et al., 2019; Fortunato et al., 2016). Different hypotheses have been considered to explain this link including sensorial deprivation, information degradation, reduction in cognitive reserve, and the presence of shared pathological pathways (i.e., microvascular damage of the brain). Interestingly, analyzing specific cognitive domains in a sample of hearing‐impaired but cognitively healthy older subjects, it has been demonstrated that hearing loss affected the episodic memory and attentive functions rather than the executive functions (Guglielmi et al., 2020). Therefore, it can be hypothesized that hearing loss would weaken the executive control over other cognitive processes by diverting attention resources toward the auditory processes; at the same time, the physiological decline in executive functions brought on by age would exacerbate, in a vicious circle, the effect of hearing loss. Accordingly, excessive cognitive load dedicated to auditory perceptual processing in everyday life causes brain structural changes and neurodegeneration relevant to the detriment of other cognitive processes (Uchida et al., 2019). At a neural level, chronic hearing loss leads to reduced activation in central auditory pathways, resulting in dysfunctional auditory–limbic connectivity and deafferentation‐induced atrophy in prefrontal brain (Rutherford et al., 2018). Moreover, several studies widely described the reciprocal relationship between late‐life anxiety and cognition, since the simultaneous presence of psychiatric symptoms and CI is very common and leads to mutual worsening, making the effectiveness of therapy increasingly difficult, what could characterize the early phases of dementia (Beaudreau & O'Hara, 2008; Rozzini et al., 2009; Sardone et al., 2020). It has been suggested that both ARHL and age‐related tinnitus affect mental health, they contribute in developing anxiety, stress, and depression (Fortunato et al., 2016). Furthermore, in individuals with hearing loss possibly associated with tinnitus, the reduced speech understanding limits socialization inducing social isolation, loneliness, and depression (Bigelow et al., 2020). Hence, the modulations of neural activity related to sensory loss appear to affect the resources required to perform higher‐level cognitive operations, supporting a resource re‐allocation of cognitive sources (Fabijańska et al., 2012; Uchida et al., 2019).

Taken together, our results demonstrated that CI in tinnitus patients could not be related to the severity or to the psychoacoustic measures of tinnitus but to the age‐related deterioration of hearing underlying psychological distress. According to this hypothesis, both ARHL and CI in tinnitus patients would be the results of a common neurodegenerative process in the aging brain. In conclusion, chronic tinnitus annoyance at THI have been correlated to the presence of anxious–depressive disorders measured by means of HADS. Age, female‐to‐male ratio, level of hearing loss, and HADS‐D and HADS‐A scores were significantly higher in patients with cognitive impairment, demonstrating that cognition especially involves the psychiatric component of affected patients regardless of tinnitus status and its subjective perception on THI. A limitation of our study is the absence of a control group that could be useful to determine the effect of tinnitus on CI. Furthermore, other aspects remain to be clarified, such as the cognitive domains predominantly involved considering that most studies conducted on cognition in tinnitus focus on one single neurocognitive function (as in our case memory), without any comparison with the other domains (Khan & Husain, 2020). Our future aim could be to explore the cognitive functions using neuropsychological test batteries for different cognitive domains.

In conclusion our findings demostrate the importance to focus on the features a of early emotional and cognitve disorders in tinnitus patients, especially in geriatric population with severe hearing impairment. We suggest the adoption of reliable tools such as THI, and nonverbal emotional and cognitive screening tests such as HADS and MMSE during the audiological counseling, in order to address patients to a specific neuropsychiatric evaluation, getting a more accurate diagnosis with a multidisciplinary approach and a more effective therapy.

## CONFLICT OF INTEREST

Authors have not received any funds from any kind of organization; the study is entirely the result of our work. We deny any kind of conflict of interest.

## AUTHOR CONTRIBUTION

Anna Rita Fetoni: Study concept and design. Tiziana Di Cesare, Stefano Settimi, Bruno Sergi Giorgia Rossi, and Camillo Marra: Data collection. Tiziana Di Cesare, Stefano Settimi, Rita Malesci, and Eugenio De Corso: Analysis and interpretation of the data. Anna Rita Fetoni, Tiziana Di Cesare, Rita Malesci, and Gaetano Paludetti: Manuscript preparation. Anna Rita Fetoni, Gaetano Paludetti, and Eugenio De Corso: Study supervision.

### Peer Review

The peer review history for this article is available at https://publons.com/publon/10.1002/brb3.2074.

## Data Availability

The data that support the findings of this study are available from the corresponding author upon reasonable request.
